# Recent Mortality Trend Reversal in Russia: Are Regions Following the Same Tempo?

**DOI:** 10.1007/s10680-017-9451-3

**Published:** 2017-11-22

**Authors:** Sergey Timonin, Inna Danilova, Evgeny Andreev, Vladimir M. Shkolnikov

**Affiliations:** 1 National Research University Higher School of Economics, Myasnitskaya St. 20, Moscow, Russia 101000; 2 Max Planck Institute for Demographic Research, Konrad-Zuse-Strasse 1, 18057 Rostock, Germany

**Keywords:** Regions of Russia, Mortality disparities, Causes of death, Decomposition

## Abstract

After several decades of negative trends and short-term fluctuations, life expectancy has been increasing in Russia since 2004. Between 2003 and 2014, the length of life rose by 6.6 years among males and by 4.6 years among females. While positive trends in life expectancy are observed in all regions of Russia, these trends are unfolding differently in different regions. First, regions entered the phase of life expectancy growth at different points in time. Second, the age- and cause-specific components of the gains in life expectancy and the number of years added vary noticeably. In this paper, we apply decomposition techniques—specifically, the stepwise replacement algorithm—to examine the age- and cause-specific components of the changes in inter-regional disparities during the current period of health improvement. The absolute inter-regional disparities in length of life, measured by the population-weighted standard deviation, decreased slightly between 2003 and 2014, from 3.3 to 3.2 years for males, and from 2.0 to 1.8 years for females. The decomposition of these small changes by ages and causes of death shows that these shifts were the result of diverse effects of mortality convergence at young and middle ages, and of mortality divergence at older ages. With respect to causes of death, the convergence is mainly attributable to external causes, while the inter-regional divergence of trends is largely determined by cardiovascular diseases. The two major cities, Moscow and Saint Petersburg, are currently pioneering mortality improvements in Russia and are making the largest contributions to the inter-regional divergence.

## Introduction

The Russian mortality crisis, which started in the mid-1960s and continued over the later decades of the twentieth century, is well documented in the literature (Field 1995; Shkolnikov et al. 1996, 2004; Shkolnikov and Cornia 2000). From 1965 to 1984 in Russia, male life expectancy decreased from 64.4 to 61.7 years, while female life expectancy stagnated, having barely risen from 73.0 to 73.8 years. Similar negative tendencies (stagnating or even decreasing life expectancy) were observed in other parts of the USSR, and (in a somewhat attenuated form) in the countries of Central Europe (Meslé 2004). The anti-alcohol campaign of 1985–1988 resulted in a rapid increase in life expectancy in Russia (Shkolnikov and Nemtsov 1997). Within a few years after the start of the campaign, male life expectancy recovered to mid-1960s levels, and female life expectancy even exceeded those levels. The abrupt termination of the anti-alcohol measures, which was followed by a sudden increase in the availability and the accessibility of alcohol, was also accompanied by a series of painful political and economic changes. The chain of these events resulted in a sharp decline in life expectancy at the beginning of the 1990s (Shkolnikov and Cornia 2000; Shkolnikov et al. 2001). In 1994, life expectancy in Russia fell to the lowest levels ever recorded in the country: to 57.4 years for males and to 71.7 years for females. After hitting these low points, life expectancy increased rapidly between 1994 and 1998; over this 4-year period, females gained 2.1 years and males gained 3.8 years. However, this upturn was short-lived. After 1998, a new period of life expectancy decline (though not as rapid as in the early 1990s) began. The year 2003 marked one more turning point, after which length of life in Russia again started to rise, and has continued to increase up to today. The year 2012 marked the first time in history that Russian life expectancy at birth (for both sexes combined) exceeded 70 years (Human Mortality Database 2016).

The current period of life expectancy improvements differs from the two previous periods in the mid-1980s and the mid-1990s in at least two ways: (a) it has lasted much longer, and is thus the longest period during which life expectancy has been continuously growing since the mid-1960s (Shkolnikov et al. 2013); and (b) the recent mortality improvements have spread to older ages (largely due to the decrease in cardiovascular mortality), whereas during the previous periods, the contributions of the older age groups to increasing life expectancy were minor (Shkolnikov et al. 2013; Grigoriev et al. 2014). Researchers who have studied these trends have argued that there is some evidence that Russia finally entered the cardiovascular revolution in the 2000s (Andreev et al. 2014; Grigoriev et al. 2014).

Within the health transition framework, it can be argued that any major innovation that introduces a new mortality regime can launch a sequence of divergence/convergence processes (Frenk et al. 1991; Vallin and Meslé 2004, 2005). Each new successive stage of the health transition starts with divergence, i.e., in the initial stages of the transition, only pioneer populations are able to implement new strategies and technologies that result in mortality reduction, while other populations lag behind; and some time later, the laggards adopt the improvements and start to catch up with the pioneers. In the majority of studies on the health transition, the processes of divergence/convergence in mortality are highlighted using inter-country comparisons (McMichael et al. 2004; Moser et al. 2005; Clark 2011; Edwards 2011; Mackenbach 2013; Goli and Arokiasamy 2014); other studies have examined the divergence/convergence process across socio-demographic groups (Shkolnikov et al. 2011; Kibele et al. 2013; Jasilionis et al. 2014). An investigation of how the shift toward a new mortality regime spreads at the sub-national level seems to be of equal importance. Analyses of divergence/convergence in mortality across space have, for example, been performed in the USA (Ezzati et al. 2008; Wang et al. 2013; Currie and Schwandt 2016), Germany (Kibele 2012), England and Wales (Bennett et al. 2015), India (Goli and Arokiasami 2013; Saikia et al. 2011), Austria (Gähter and Theurl 2011), and Spain (Montero-Granados et al. 2007).

A few studies on Russia have looked at the patterns of regional mortality inequality and the changes in these patterns over time. A southwest to northeast geographical mortality gradient—with the lowest life expectancy in the northeast and the highest life expectancy in the southwest—was detected by Andreev (1979) and Shkolnikov (1987) and was re-confirmed by later studies (Shkolnikov and Vassin 1994; Vassin and Costello 1997; Vallin et al. 2005; Kvasha and Kharkova 2011). The observed geographical variation was linked to general socioeconomic development levels, natural conditions, and alcohol consumption levels. These studies found that external causes and cardiovascular diseases have been the two major drivers of the regional variation in causes of death in Russia. It has also been shown that the mortality fluctuations that occurred in Russia during the 1990s were negatively associated with the changes in the variation across regions (Ivaschenko 2005).

In the context of the current study, we aim to answer two questions:

1. How have the regional disparities in mortality changed over the recent period of health improvements in Russia (from 2004 onward)?
2. And, what were the major age- and cause-specific components of these changes?

To answer these questions, we start by examining regional disparities in life expectancy in Russia over the whole period for which regional mortality data are available. This approach allows us to quantify the link between the periods of divergence/convergence in regional mortality and the changes in overall life expectancy at the national level. The population-weighted standard deviation of regional life expectancies is used as a measure of inter-regional disparities. We then focus on the components of the recent shift in cross-regional variation in life expectancy, applying the stepwise replacement algorithm to decompose by ages and causes of death changes in the average population-weighted life expectancy, and in the standard deviation of life expectancy across regions.

In light of previous findings, we expect to observe an overall pattern of convergence; and to find that the reduction in mortality from external, alcohol-related, and acute cardiovascular conditions at young and middle ages has been playing a large role in this trend. We may, however, also see some deviations from this characteristic pattern.

## Data and Methods

### Initial Data

The initial data on death counts by age, sex, and cause of death and the population estimates are provided by the Russian Federal State Statistics Service (Rosstat) for each unit of the highest level of Russia’s administrative division (henceforth referred to as “regions”).^1^ For the years before 1989, mortality estimates at the regional level can be performed only for the 2-year periods around the USSR censuses of 1970 and 1979; for the years since 1989, these data are available annually (1989–2014). All-cause death counts and population estimates by sex are available for 1-year age groups, with the final group being an open-ended interval of 100 years and older; while the cause-specific mortality data are available for 5-year age groups only (0, 1–4, 5–9, …, 85+).

For the cause-specific mortality estimates (for the period 2003–2014), we rely on the data on causes of deaths as identified using the Russian Abridged Classification, which is based on ICD-10 (the Revision of 1999). We aggregated the items of the Russian Abridged Classification into seven broad categories, namely cardiovascular diseases, neoplasms, external causes, infectious diseases, respiratory diseases, digestive diseases, and all other causes of death (for more details, see Table 1 in “Appendix”). Ill-defined codes were redistributed among other groups of causes of death. More details on treating ill-defined codes can be also found in “Appendix” (Table 1).

### Issues of Data Quality

**Fig. 1:**
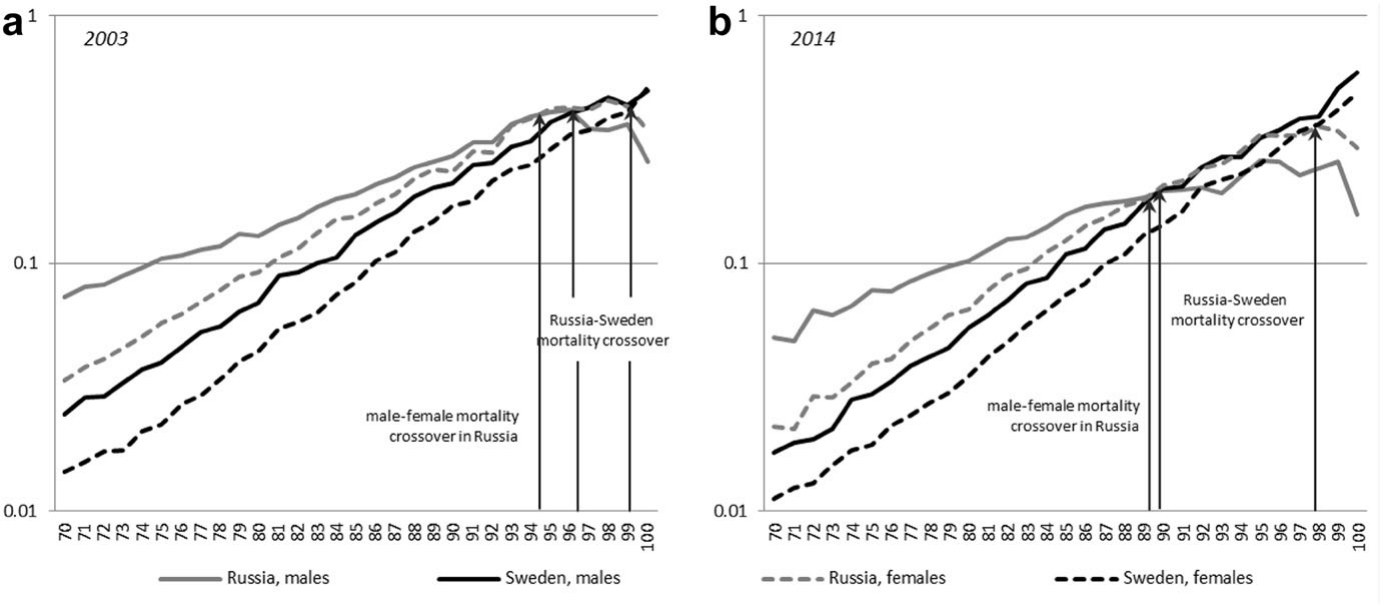
Mortality rates at ages 70+ in Russia and Sweden, by sex, 2003 (**a**) and 2014 (**b**) (logarithmic scale). *Source*: Human Mortality Database (2016)

Quality problems affect mortality data at advanced ages, even in highly developed countries (Jdanov et al. 2005; Thatcher et al. 2002). As there are considerable concerns about the quality of Russian data at ages 80 and older (Human Mortality Database 2016), we treat these mortality data with caution. Figure 1 compares male and female death rates in Russia and Sweden^2^ in 2003 and 2014. The figure highlights the implausibly low mortality levels at advanced ages and the male–female mortality crossovers in Russia, as these patterns may be indicative of data quality problems (Fig. 1a, b).

There is no evidence of a systematic under-registration of deaths in Russia, and death registration in Russia is regarded as “complete” by the WHO (2014). Therefore, the discrepancies in mortality trends at old ages could indicate problems with age reporting, such as the exaggeration of an individual’s age at death or of the ages of the enumerated population (Human Mortality Database 2016; Andreev 2012; Anderson and Silver 1997). Another possible explanation for these discrepancies is the presence of a so-called “statistically immortal population,” a distortion that can occur when individuals who die abroad continue to be counted in the population (Jdanov et al. 2005; Scholz 2007) (this problem can be even more relevant for estimating mortality at the sub-national level). The discrepancies in the mortality estimations could also be attributable to the “double counting” of the population at the last census (Andreev 2012; Mkrtchyan 2012).

Figure 1 suggests that the aforementioned inconsistencies became more pronounced between 2003 and 2014. It thus appears that mortality statistics at older ages deteriorated somewhat during this period, which could place limits on the interpretation of the results of our analysis. However, the principal findings of our study should not be substantially affected by these discrepancies. As Fig. 1 shows, mortality understatement is observed in Russia starting at ages 85–90 among males and at ages 90–95 among females. As the mean ages at death in Russia of 65.3 years for males and 76.5 years for females in 2014 are still considerably below these ages, the regional life expectancy estimates are not greatly distorted by the aforementioned inconsistencies. However, all of the analytical results specifically related to older ages are questionable (Further limitations of our study are discussed in Sect. 4.1).

### Methods

#### Measurement of Inter-regional Disparities

Based on the data on death counts and population estimates, we computed age-specific mortality rates (*m*_*x*_) and derived abridged life tables for the male and the female populations of each region (*i* = 1,…, *k*) for all available time points.^3^ The aggregate measure for *k* regions at time *t* is the population-weighted average life expectancy^4^:

1 $$\overline{{e_{0} \left( t \right)}} = \mathop \sum \limits_{i} \pi_{i} \left( t \right)e_{0,i} \left( t \right),$$

with the population weights being $\pi_{i} \left( t \right) = \frac{{\mathop \sum \nolimits_{x} p_{x,i} \left( t \right)}}{{\mathop \sum \nolimits_{i} \mathop \sum \nolimits_{x} p_{ x,i} \left( t \right)}}$, where $p_{x,i } \left( t \right)$ is the population at age group *x* in region *i* in year *t*, and *e*_0,*i*_(*t*) is the life expectancy at birth in regions *i* in year *t*.

Following Edwards and Tuljapurkar (2005) and Timonin et al. (2016), the population-weighted standard deviation, counted in years, is used as a measure of lifetime disparities across the regions:

2 $${\text{wSD}}\left( {e_{o} \left( t \right)} \right) = \left[ {\mathop \sum \limits_{i} \pi_{i} \left( t \right)\left[ {e_{0,i} \left( t \right) - \overline{{e_{0} \left( t \right)}} } \right]^{2} } \right]^{1/2} .$$

We use the population-weighted (as opposed to unweighted) standard deviation as a measure for the sizes of the inter-regional disparities because it takes into account not only the life expectancy differences between regions, but also how many people were exposed to a higher or a lower death hazard in a particular region. However, the disadvantage of using this metric is that it has a low degree of sensitivity to mortality changes in regions with small populations. The issues associated with weighting disparity measures are additionally discussed in the sensitivity analysis Sect. 3.4.

To test the divergence/convergence process in regional trends of life expectancy, we refer to the concepts of sigma- and beta-convergence. *Sigma*-*convergence* is observed when the disparity measure of a certain indicator decreases over time. In our analysis, the presence of sigma-convergence in life expectancy across regions can be expressed as follows:

3 $${\text{wSD}} \left( {e_{o} \left( {t_{0} } \right)} \right) > {\text{wSD}} \left( {e_{o} \left( t \right)} \right).$$

To test for *beta*-*convergence,* we apply a weighted linear regression model that predicts how changes in the regional values of life expectancy between 2003 and 2014 depend on the initial level of life expectancy in the year 2003. We solve regression equation:

4 $$\Delta e_{i,o} \left( {t_{0;} t} \right) = \alpha + \beta \cdot e_{i,o} \left( {t_{0} } \right)_{i} \,+ \,\varepsilon_{i} ,$$

minimizing the weighted sum of squared residuals:

$$\mathop \sum \limits_{i = 1}^{k} \pi_{i} \left( {t_{0} } \right) \cdot \left( {\Delta e_{i,o} \left( {t_{0;} t} \right) - \left( {\alpha + \beta \cdot e_{i,o} \left( {t_{0} } \right)_{i} } \right)} \right)^{2} ,$$

where $\Delta e_{i,o} \left( {t_{0;} t} \right)$ is the gain in life expectancy at birth between 2003 and 2014 in region *i.*

The significantly negative slope-coefficient $\beta$ indicates the presence of the beta-convergence.

#### Decomposition of Inter-regional Disparities in Life Expectancy

In order to gain a better understanding of the convergence/divergence process at the sub-national level, we suggest decomposing the changes in the population-weighted life expectancy and standard deviation measures by ages and causes of death. The conventional decomposition methods introduced by Andreev (1982), Pollard (1982), Arriaga (1984), and Pressat (1985) are not suitable for performing this task, as they can only be used to decompose a change in life expectancy in a single population between two time points or between two populations at one point in time. We instead employ the general stepwise replacement algorithm that allows us to decompose measures based on several populations (Andreev et al. 2002). In Timonin et al. (2016), this method was used to decompose changes in between- and within-group variance in life expectancy for a set of developed countries. However, unlike in the current study, information on causes of death was not included in the decomposing procedure.

Let $m_{x,j, i}$ be the age- (0, 1–4, 5–9,…, 85+) and cause-specific (*j* = 1,…, *m*) mortality rate in region *i*. Applying the stepwise replacement to the set of regional populations, the age- and cause-specific components, as well as their mortality and population composition parts (M- and P-effects), can be obtained by running a sequence of replacements of age- and cause-specific mortality rates in each region in the year *t*_0_, $m_{x,j,i} \left( {t_{0} } \right)$, by the corresponding rates $m_{x,j,i} \left( t \right)$ in the year *t,* and vice versa. The replacement runs in the ascending order across all ages (from the youngest age zero to the oldest age 85+) and across all possible combinations of causes of death under study.

The component corresponding to a change in each age group due to the specific cause of death is a sum of the mortality and the population composition parts (M- and P-effects):

5 $$\Delta^{{\left[ {x,j} \right]}} = \Delta_{\text{M}}^{{\left[ {x,j} \right]}} + \Delta_{\text{P}}^{{\left[ {x,j} \right]}} .$$

The mortality-related part (M-effect) of the total change refers to differences in mortality and mortality changes across regions, while the population-related part (P-effect) reflects the changes in the population size and structure of the regions. Since the changes in the regions’ population weights over 2003–2014 are minor, we proceed in our analysis by examining the M-effects only. Finally, the total change in the population-weighted standard deviation between times *t*_0_ and *t* is:

6 $${\text{wSD}}\left( {e_{0} \left( t \right)} \right) - {\text{wSD}}\left( {e_{0} (t_{0} )} \right) = \mathop \sum \limits_{x} \Delta^{{\left[ {x,j} \right]}}$$

## Results

### Life Expectancy Fluctuations and Trends in Inter-regional Disparities

We start our analysis by examining the links between the value of life expectancy in Russia, and how it varies at the sub-national level. Summary statistics on changes in life expectancy and inter-regional disparities are presented in Table 2 (in “Appendix”).

**Fig. 2:**
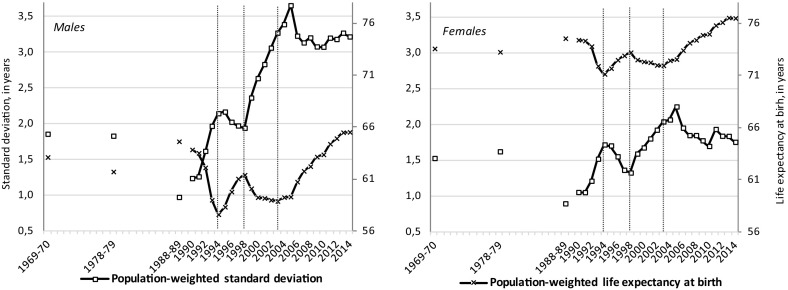
Trends in population-weighted life expectancy at birth and population-weighted standard deviation in Russia, by sex; 1969–1970, 1978–1979, 1988–1989, and 1990–2014 (in years). *Note*: Vertical dash lines show the time points of the changes in trends in life expectancy at the national level. *Source*: Own calculation based on data provided by Rosstat

The inverse correlation between life expectancy and wSD in Russia is especially pronounced, and corresponds to the short-term fluctuations in life expectancy observed in the 1990s and the 2000s (Fig. 2). The turning points in the life expectancy trend coincide with the turning points in the wSD trend.

The lowest observed wSD within the years available for analysis was recorded just after the anti-alcohol campaign, and its value was almost the same for males and females (1.0 and 0.9 years, respectively). This arguably proves that alcohol-related mortality played a crucial role in the time trend changes and the regional variation in life expectancy in Russia. The highest values of wSD were reached in 2005 (3.7 years for males and 2.2 years for females). This development was attributable to the opposing trends in life expectancy at the regional level in the early 2000s. While the life expectancy increase started in some regions of Russia at the very beginning of the 2000s, in other regions life expectancy continued to decline until 2005 (Table 3 in “Appendix”). Thus, the previously identified inverse correlation between life expectancy and wSD cannot be directly transferred to the most recent period in Russia, during which health was improving. First, despite the slight increase in life expectancy at the national level in Russia between 2003 and 2005, the inter-regional variation did not decrease, but continued to rise rapidly through that period. Second, after 2005, wSD decreased to the level observed prior to the recent health improvements, and has, with minor fluctuations, remained unchanged since then.

### Life Expectancy Increase in 2003–2014 and Its Regional Patterns

In this section, we focus on the recent phase of increasing life expectancy that started in Russia after 2003. Over the 2003–2014 period, the population-weighted life expectancy at birth increased from 58.9 to 65.5 years for males, and from 71.9 to 76.5 years for females. This increase was mainly due to mortality-related changes (6.65 years for males and 4.54 years for females), and to a much smaller extent to the changes in the population distribution across regions (0.18 years for males and 0.10 years for females).

The initial levels of life expectancy in 2003 and the gains in life expectancy between 2003 and 2014 across regions are negatively correlated at the regional level with the weighted Pearson’s coefficient of − 0.319 (*p* = 0.002) for males and − 0.559 (*p* < 0.001) for females. The beta-coefficients calculated from the weighted regression model were − 0.133 (95% CI − 0.222, − 0.440; *p* = 0.004) for males and − 0.267 (95% CI − 0.356, − 0.177; *p* < 0.001) for females, which indicates that the regions with low initial levels of life expectancy experienced more rapid increases, and were thus catching up to the regions with better starting positions.

**Fig. 3:**
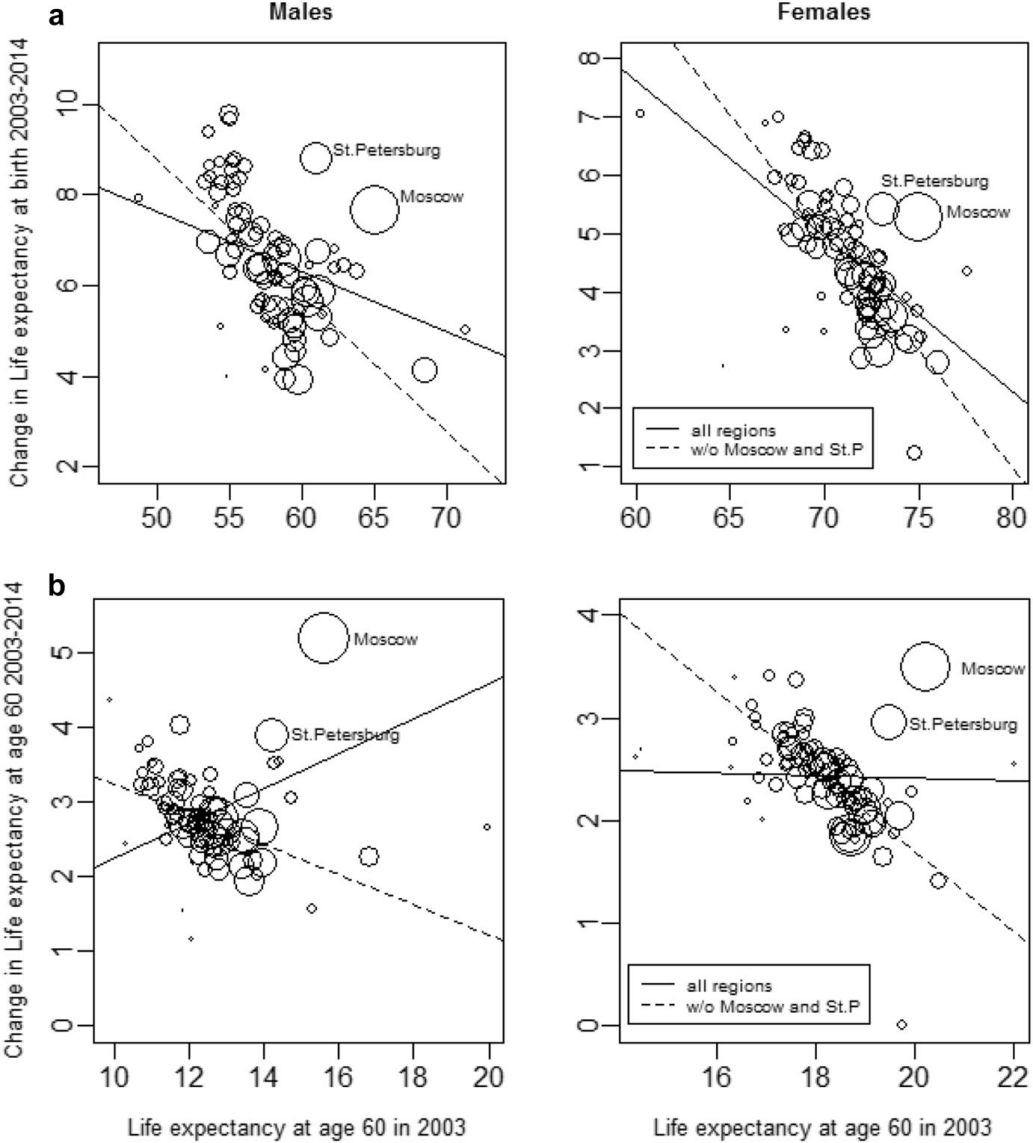
Correlation between the regional levels in life expectancy at birth (**a**—upper panel) and at age 60 (**b**—lower panel) in 2003 and the corresponding changes between 2003 and 2014, by sex (in years). *Note*: The trend line is estimated by weighted OLS regression; the circle size indicates the population in each region in the year 2003. *Source*: Own calculation based on data provided by Rosstat

Although a correlation between the initial levels of life expectancy at birth and the changes in 2003–2014 is noticeable (Fig. 3, panel a), some regions deviated rather significantly from the general pattern. The cities of Moscow and Saint Petersburg are among the most obvious outliers. In these two regions, the increases in life expectancy between 2003 and 2014 were much larger than would be expected given their starting positions. As Moscow and Saint Petersburg have large populations, these deviations greatly affect the overall relationship between the starting levels and the increments across the regions and have large effects on the weighted averages and standard deviations. When we analyze the correlation between the life expectancies at age 60 and their changes, the outlier positions of Moscow and Saint Petersburg become even more pronounced (Fig. 3, panel b). The inclusion or the exclusion of these two major cities significantly changes the statistical relationship between the starting levels and the changes in regional life expectancies, especially at ages 60 years.

The decomposition of the mortality-related changes in life expectancy in Russia by regions and age groups proves that all of the regions contribute positively, and shows the size of each contribution which depends both on the gains in life expectancy in a particular region, and on its population size. The age patterns of mortality reduction also differ across regions (for more details see Table 4 in “Appendix”).

**Fig. 4:**
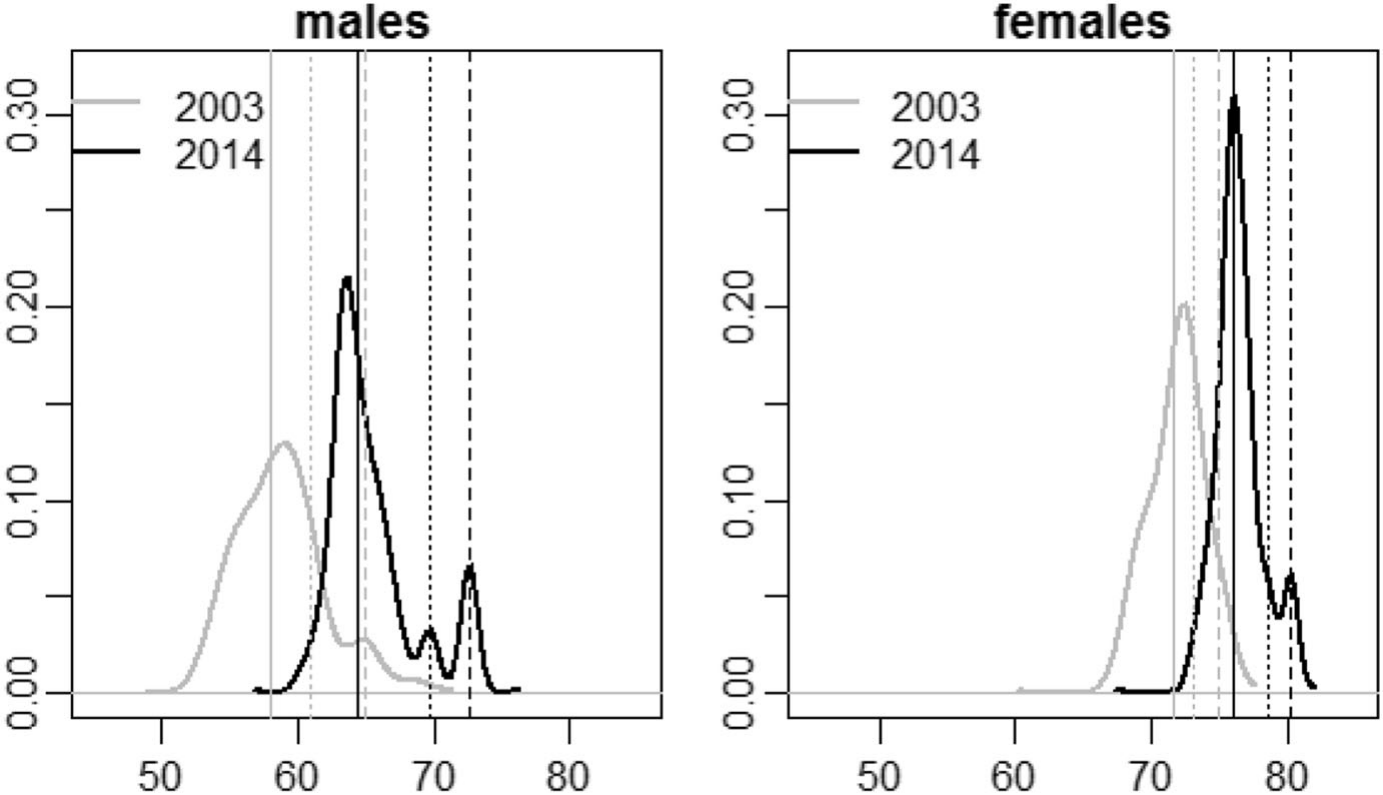
Distributions of life expectancies at birth across Russian regions. *Note*: The vertical solid lines indicate the value of life expectancy in Russia, excluding the cities of Moscow and Saint Petersburg; dashed lines—in the city of Moscow; dotted lines—in the city of Saint Petersburg. *Source*: Own calculation based on data provided by Rosstat

Figure 4 shows the weighted distributions of life expectancy at birth across the Russian regions in 2003 and 2014. At a starting point, the favorable positions of Moscow and Saint Petersburg relative to the positions of the rest of Russia’s regions were already recognizable, especially among the male population. However, by 2014, the advantage of being a Muscovite or a Petersburger in terms of life expectancy gains had increased. In 2003, males in Moscow lived 7.1 years longer and males in Saint Petersburg lived 3.0 years longer than males in Russia as a whole. By 2014, these gaps had grown to 8.3 and 5.3 years. The corresponding gaps for females were + 3.5 years in Moscow and + 1.6 years in Saint Petersburg in 2003, and increased up to + 4.3 years in Moscow and + 2.6 years in Saint Petersburg in 2014.

The right skews of the distributions are caused not only by these two cities, but also by some low mortality republics of North Caucasus. However, because these republics have small populations, their contributions to inequality across Russia are insignificant compared to those of Moscow and Saint Petersburg. If we take into account only the “larger” (left-hand side) parts of the distributions, we can see that the distributions have narrowed. This leads us to conclude that there has been both (1) a divergence of Moscow and Saint Petersburg on the one hand and the rest of Russia on the other, and (2) a convergence of the regions that constitute “the rest of Russia.” We examine it further by decomposing changes in life expectancy and population-weighted standard deviation by ages and causes of death.

### Age- and Cause-Specific Components of Changes in Life Expectancy and Inter-regional Disparities

**Fig. 5:**
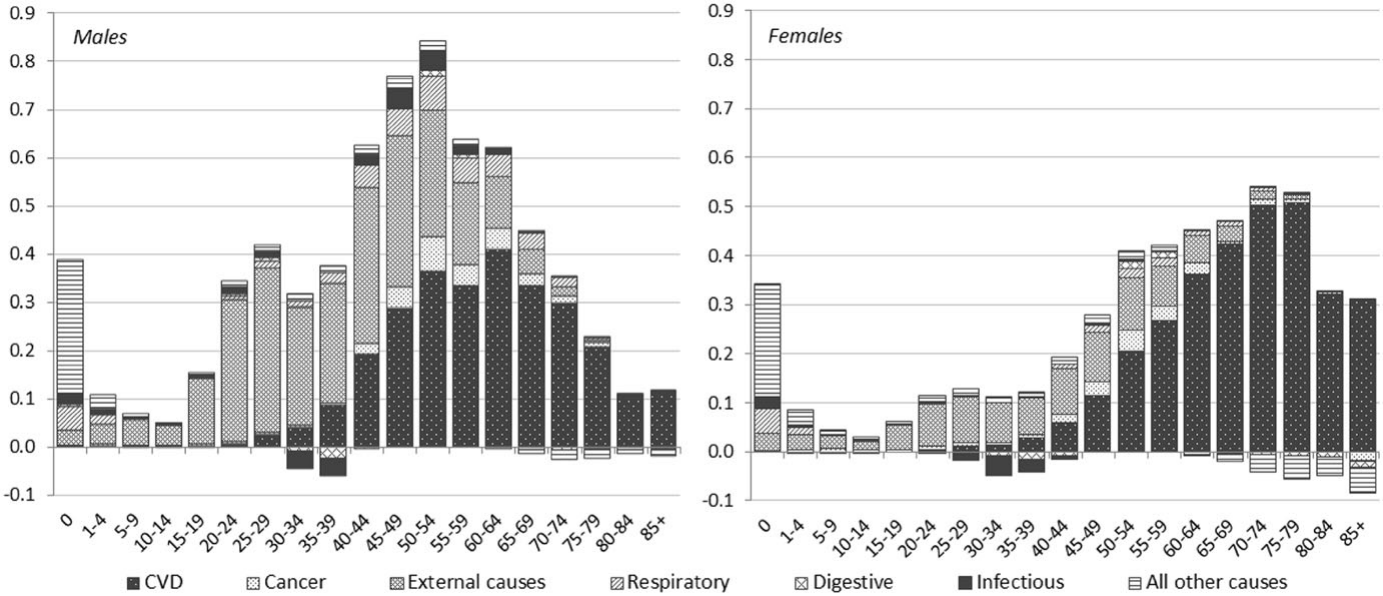
Age- and cause-specific components of the changes in population-weighted average life expectancy at birth, by sex, 2003–2014 (in years). *Source*: Own calculation based on data provided by Rosstat

Figure 5 presents the results of the decomposition by ages and causes of death. With respect to age, the increase in the overall life expectancy was attributable to the reduction in mortality at all ages, and particularly at working ages for males, and at older ages for females. Looking at the causes of death, we can see that the reduction in mortality from cardiovascular diseases (CVD) and from external causes of death contributed almost equally and most significantly to increasing life expectancy among males (about 40% each). Among females, the reduction in mortality was mainly attributable to the decline in CVD (accounting for more than 70% of the total increase in life expectancy), and secondarily to the decrease in external causes (20%). The negative contributions of infectious and digestive diseases at ages 30–40 and of the group of “all other causes” at advanced ages are also worth noting.

**Fig. 6:**
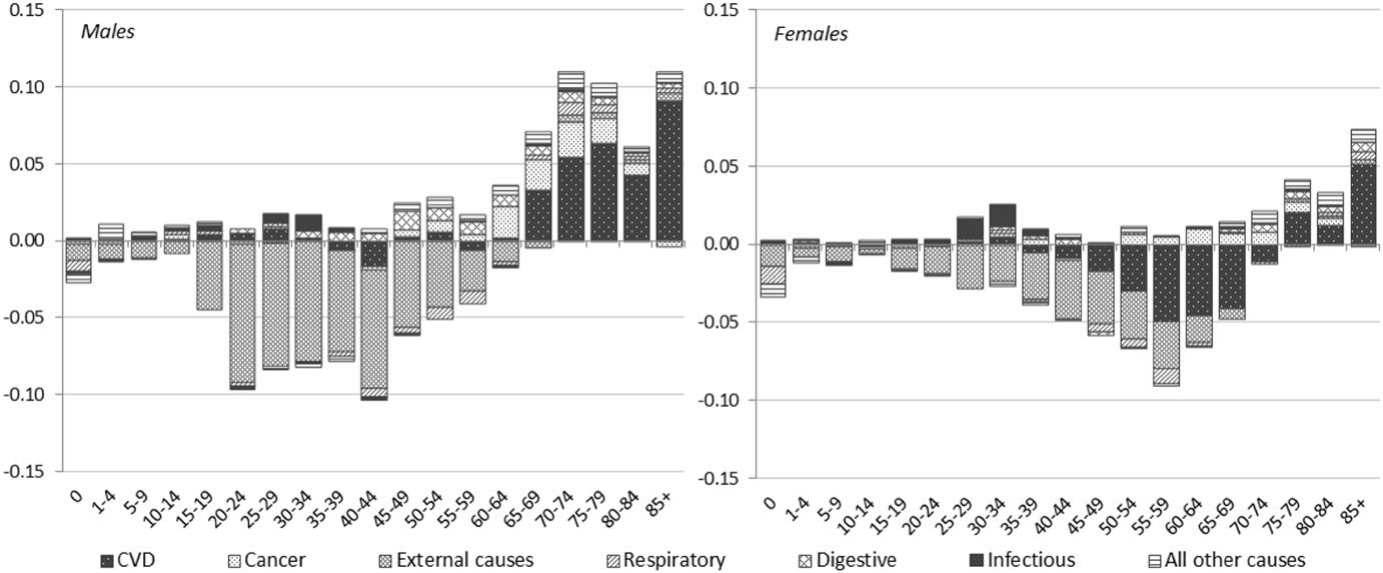
Age- and cause-specific components of the mortality-related changes in standard deviation, by sex, 2003–2014 (in years). *Source*: Own calculation based on data provided by Rosstat

As we mentioned above, the total differences in wSD between the two time points, 2003 and 2014, turn out to be almost negligible (− 0.1 for males and − 0.3 years for females). However, the decomposition of the change in wSD shows the varying contributions of different ages and causes of death to the divergence/convergence process (Fig. 6).^5^

We can see the distinct shift away from the convergent contributions at younger ages toward the divergent contributions at older ages. In other words, mortality changes at young and middle ages caused the regions to converge, but mortality dynamics at old ages caused the regions to diverge. The age boundary of the convergence-to-divergence shift is ages 70–74 for females and ages 60–64 for males.

As we mentioned above, reductions in mortality from CVD and external causes of death contributed the most to the overall life expectancy increase in Russia (about 80% for males and 90% for females). However, the patterns of the decline in mortality from these two cause-of-death groups differed from each other and varied significantly across regions. Changes in the rates of deaths from external causes pushed the regions to converge, while changes in the rates of death from CVD were the main drivers of the divergence at the sub-national level, but varied with respect to sex and age (Fig. 6). For males, it appears that CVD mortality had almost no effect at middle ages, and that the divergence process for this cause of death occurred at ages 65 and older. These findings suggest that further reductions in CVD mortality could have huge effects on life expectancy among males at the sub-national level in Russia. For females, the situation is different; we see a convergent effect of CVD mortality at ages 45–74, and then a shift toward divergence at advanced ages.

Our analysis shows substantial differences in the age- and the cause-specific contributions made by males and females to the increase in average life expectancy and to the changes in inter-regional disparities between 2003 and 2014. When analyzing these results, it is important to bear in mind the huge gender gap in life expectancy in Russia, which was 13.3 years in 2003 and 11.2 in 2014. In general, this gap implies that males and females in Russia represent two sub-populations with rather different mortality curves and indicates that mortality decline started from very different levels in males and females, as male mortality is characterized by much higher rates of death at young adult and middle ages. We would therefore expect to find that even similar changes in mortality rates in males and females would contribute differently to the changes in life expectancies and inter-regional disparities.

### Sensitivity Analysis

**Fig. 7:**
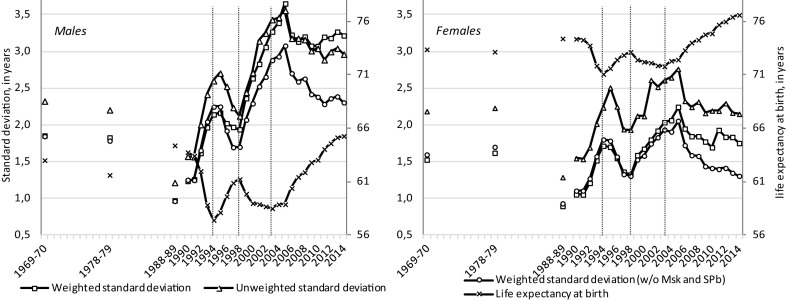
Trends in life expectancy at birth in Russia and standard deviation: weighted by regional populations (all regions and excluding Moscow and Saint Petersburg) and unweighted, by sex; 1969(70), 1978(79), 1988(89), and 1990–2014. *Note*: Vertical dash lines show the time points of the changes in the trends in life expectancy at the national level. *Source*: Own calculation based on data provided by Rosstat

**Fig. 8:**
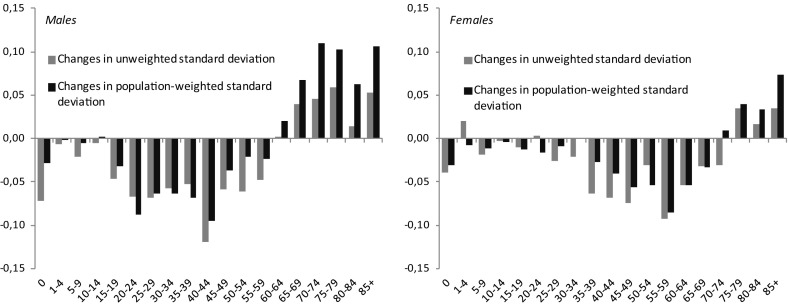
Age components of the changes in population-weighted and unweighted standard deviations, by sex, 2003–2014 (in years). *Source*: Own calculation based on data provided by Rosstat

We examined trends in inter-regional life expectancy disparities using a measure of population-weighted standard deviation (wSD). We chose to use the standard deviation metric for several reasons: (a) it is a conventional measure of mathematical statistics, which can be easily understood by non-specialists; (b) it is counted in years and can be easily compared to the life expectancy measure; and (c) it generates results similar to those based on more comprehensive measures of inequality. We selected the population-weighted measure rather than the unweighted measure because we preferred to use a public health-oriented measure that could reflect the sizes of populations exposed to different levels of the risk of death. A disadvantage of using this measure was, however, its low sensitivity to mortality changes in regions with small populations. To address this issue, we additionally calculated the unweighted standard deviation for the entire period under study. Figure 7 in “Appendix” shows that the trend in the unweighted standard deviation is quite similar to the trend in the weighted metrics. However, because of the effects produced by the regions with small populations, most of which experienced higher mortality, the magnitude of the unweighted measure is generally higher (particularly for women). From 2003 to 2014, the age- and sex-specific patterns of changes in the unweighted standard deviation do not differ much from the patterns of changes in the weighted measure, with a few minor exceptions: the degree of convergence at young and middle ages is higher, and the degree of divergence at older ages is lower when we decompose the changes in the unweighted standard deviation (Fig. 8 in “Appendix”). Consequently, the total degree of convergence in life expectancy among the Russian regions becomes greater when the sizes of populations are not taken into account.

**Fig. 9:**
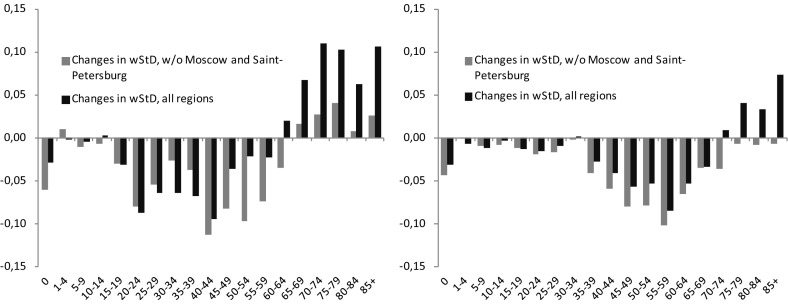
Age components of the changes in population-weighted standard deviation: all regions and excluding Moscow and Saint Petersburg, by sex, 2003–2014 (in years). *Source*: Own calculation based on data provided by Rosstat

In addition, the extent to which the results of our analysis depend on Moscow and Saint Petersburg—two federal cities with a combined population size of 17.4 million, or 12.1% of the population of Russia—is also of interest. As Fig. 7 in “Appendix” shows, changes in life expectancies in Moscow and Saint Petersburg did not have large effects on inter-regional inequalities until the end of the 1990s for males, and until the beginning of the 2000s for females. In the period that followed, the contributions of the two federal cities caused inter-regional disparities to grow, particularly during the most recent phase of health improvements in Russia. Between 2003 and 2014, the population-weighted standard deviation would have decreased by 0.58 years for males and by 0.63 years for females if Moscow and Saint Petersburg had been excluded. The exclusion of these two cities would have considerably diminished the recent contributions of older ages to inter-regional divergence and would have emphasized the contributions of younger adult ages to inter-regional convergence (Fig. 9 in “Appendix”).

## Discussion

The current increase in life expectancy in Russia can be attributed to two major trends: (1) a recovery in life expectancy growth following a mortality crisis that was especially pronounced among people of working ages, as members of this age group were disproportionately affected by earlier declines; and (2) improvements in health care and risk factors that led to a decrease in cardiovascular mortality at older ages. Previous studies have discussed the question of whether the second trend signals the beginning of a cardiovascular revolution in Russia (Shkolnikov et al. 2013, 2014; Andreev et al. 2014; Grigoriev et al. 2014). By 2014, this trend had become even more pronounced and was especially noticeable in the female population, as reductions in female cardiovascular mortality, especially at older ages, contributed 3.13 years (68%) to improvements in life expectancy. The decline in cardiovascular mortality also played a significant role in the growth in male life expectancy, although the contribution of this factor at older ages was smaller among males than among females.

However, improving population health is rather difficult without achieving health equity, i.e., ensuring that everyone has the opportunity to reach the highest possible level of health. Large and increasing differences in inter-regional mortality indicate that the distribution of strategic resources is out of balance, leaving parts of the population with lower levels of health than they should be able to attain.

Despite significant fluctuations, an upward movement of within-country variation in life expectancy can be seen in Russia since the end of the 1980s. Inter-regional inequalities have been reduced somewhat since the beginning of the 2000s, but are still higher than they were in the 1970s and the 1980s; and are even higher than they were during the 1990s, when there were large mortality fluctuations. The upward trend in life expectancy disparities across the country likely reflects the increased inequalities across sub-populations for a range of factors associated with mortality risks (Galbraith et al. 2004; Popovich et al. 2011; Akhmedjonov et al. 2013; Lehmann and Silvagni 2013).

If we focus more narrowly on the recent period in which health was improving in Russia (2003–2014), short-term intervals of both divergence and convergence between the regions can be observed at various time points. At the very beginning, between 2003 and 2005, the variation in regional levels of life expectancy was increasing due to the non-simultaneous starting points of mortality reductions across the regions. The inter-regional mortality inequalities started to decline after 2005, but this trend was interrupted in 2010. Although life expectancy has continued to increase for Russia as a whole, this positive trend is no longer accompanied by mortality convergence at the sub-national level. Thus, the inverse relationship between life expectancy at the national level and the variation in life expectancy across sub-populations, which was rather evident in the 1990s, has weakened.

The decomposition of the changes in the inter-regional disparity allows distinguishing two further trends within the framework of divergence/convergence. On the one hand, we see a trend toward convergence among males under age 60 and among females under age 70, which indicates that the regions that previously had relatively high mortality at these ages have been catching up to the regions with lower mortality. Thus, the recovery of growth after the mortality crisis is now reaching the convergence stage. On the other hand, the disparities at older ages have increased, which indicates that if the cardiovascular revolution has started in Russia, it has affected the regions unevenly. Currently, we can see a large degree of divergence, with some regions representing the “pioneers” of improvements, and other regions representing the “laggards.”

Our analysis showed a widening gap in life expectancy between two major Russian cities (Moscow and Saint Petersburg) and the majority of the other Russian regions. A number of factors might contribute to this gap. First, as we mentioned above, the extent of the biases in the mortality statistics is probably greater in these regions due to the difficulties in obtaining the correct exposures under high migration flows. However, such a large gap cannot be explained solely by statistical inconsistencies. The cities of Moscow and Saint Petersburg differ from the other regions with respect to a number of parameters. While the other regions encompass an aggregation of various types of settlements, ranging from rural settlements to large cities, Moscow and Saint Petersburg are both metropolises inhabited almost exclusively by an urban population.^6^ The characteristics of the residents of these cities, including their income and educational levels, differ considerably from those of residents of the rest of Russia [e.g., 46% of Muscovites and 40% of Petersburgers aged 25 and older have higher education, compared to 23% of the rest of the Russian population (FSSS 2012)]. The importance of socioeconomic determinants in explaining mortality differences in Russia has been demonstrated by previous studies (Shkolnikov et al. 1998; Shkolnikov and Andreev 2000; Perlman and Bobak 2008; Bessudnov et al. 2011).

In addition to the specific socioeconomic characteristics that may influence health behavior, Muscovites and Petersburgers likely also have better access to primary health care and advanced medical treatment, including high-technology medical care. Well-equipped facilities and highly qualified medical personnel are concentrated in specialized medical centers in these two cities. Although residents of all regions of Russia can be treated in these centers, Muscovites and Petersburgers have easier access to these medical facilities. Moreover, when seeking treatment or diagnostic procedures at these facilities, residents of Moscow and Saint Petersburg do not incur the additional costs in terms of time or money faced by residents of other regions.

In the examples of Moscow and Saint Petersburg, we see a possible pure effect of the advantages of being a resident of a metropolitan city in Russia. The residents of other megalopolises of Russia may also realize benefits (in terms of years of life expectancy) from living in big cities. But because these other big cities are not isolated in the statistics from the regions in which they are located, we cannot see these effects when analyzing mortality data at the regional level. Further investigations at a more disaggregated (district) level or at an individual level are needed to analyze the link between the place of residence and mortality in Russia.

### Limitations of the Study and Future Work

A few important limitations of our study need to be acknowledged. We have already mentioned that mortality statistics at advanced ages are somewhat biased in Russia (Sect. 2.3.1). These inaccuracies appear to be more pronounced for certain regions, including for the Muslim republics of North Caucasus and the city of Moscow (Andreev 2012). To account for the possibility that our results could be affected by inaccurate population estimates at older ages, we tested them by fitting mortality at ages 80+ according to a Gompertz curve. Though the calculations based on fitted and observed death rates gave us slightly different results, the overall picture and our main findings have not changed. Nevertheless, the results of our analysis for advanced ages should be interpreted with a reasonable degree of caution.

Another possible source of bias is the system of death registration in Russia. All medical death certificates in Russia should be registered with the offices of the Registration of Acts of Civil Status (ZAGS), (usually) within 3 days of the death. According to the Russian Federal Law on Acts of Civil Status, the civil registration of a death can be performed either at the place of death or at the deceased’s permanent residence. Thus, the family members of the deceased can deliver the medical death certificate and register the death either at the ZAGS office located in the region of the deceased’s permanent residence, or at the ZAGS office located in the region where the death occurred. Both the place of residence and the place of death are listed on the medical death certificate, but the national statistical service assigns each death to the place of the deceased’s ZAGS registration. Thus, the regional death counts can include the deaths of nonresidents (if the death was registered by the place of death) and can conversely omit the deaths of residents that occurred in other regions. This system of registration could result in discrepancies between the numerator and the denominator of mortality rates at the regional level, as the death counts may not correspond to the population at risk.

The biases caused by assigning deaths to the statistics of the region where the ZAGS registration took place should be greater for the regions with bigger incoming and/or outgoing migrant flows. Thus, this problem is probably most acute for the cities of Moscow and Saint Petersburg (which are included in the highest level of administrative division of Russia as separate federal subjects), and for the Moscow and Leningrad oblasts surrounding these cities. There are huge commuting flows between the city of Moscow and the Moscow oblast, and between the city of Saint Petersburg and the Leningrad oblast. These movements can result in very large fluctuations in the de facto population within short periods of time (Makhrova et al. 2012; Makhrova and Kirillov 2015).

In addition to these within-agglomeration “migrants,” the Moscow and Saint Petersburg agglomerations attract massive flows of migrants from other regions of Russia and from other countries. Some of these individuals are not officially registered as permanent residents of Moscow/Saint Petersburg. On the other hand, many Muscovites (these arguments should be valid for Petersburgers as well) who move to other regions of Russia or abroad for a long period of time may neglect to notify authorities of their absence, as retaining permanent residence in Moscow may provide them with certain benefits (e.g., easier access to medical facilities or certain social subsidies) (Florinskaya et al. 2015).

Despite these limitations, it is worth noting that between 2003 and 2014, there were no changes in the methodology used in the collection of vital statistics in Russia. Thus, the aforementioned issues did not arise recently and likely had the same effects in 2014 as in 2003. However, such discrepancies could be more significant if the number of short-term migrations (that are not accompanied by official registration and a change in the place of residence) increased during the period. We would also like to emphasize that the data we used in our study are produced by the Russian Federal State Statistics Service, and that these data serve as a source of information for developing health care strategies by both the federal and the regional governments.

The current study focused on identifying the changes in inter-regional disparities in life expectancy over time in Russia, and on decomposing these changes into age- and cause-specific components. However, the results obtained lead to new research questions that should be addressed in the future. In the current study, we relied on routine mortality data at the regional level, which is the highest level of administrative division in Russia. These data are the only territorial data that are available annually. However, the invisible differences in mortality within these regions may also be significant. The favorable mortality patterns in Moscow and Saint Petersburg suggest that it would be interesting to examine mortality differences between major cities, medium-size cities, smaller townships, and rural areas. It is very likely that the within-regional distribution of the population by type of settlement explains some proportion of inter-regional disparities.

The other factors also need to be considered as possible contributors to inter-regional inequalities in life expectancy in Russia. In particular, the uneven declines in mortality from CVD among elderly Russians might be attributable to the inter-regional inequalities in access to medical services and in levels of welfare among elderly people. These patterns may be seen as worrying signs of growing inter-regional differences in the financing, the productivity, and the quality of cardiovascular medicine. However, the factors implicated in the trend toward divergence need to be further investigated by future studies using data that contain more detail.

## Conclusion

Our study allowed us to distinguish various processes hidden behind the current growth in life expectancy in Russia and its representation at the sub-national level. We found that the almost unchanged levels of inter-regional disparities in the length of life after 2003 are the result of compensating divergence/convergence effects. Changes in mortality among males under age 60 and among females under age 70 led to convergence across the regions, whereas mortality dynamics at older ages led to divergence at sub-national level. With respect to causes of death, mortality reduction from external and ill-defined causes contributed to convergence across the regions; the effects of regional changes in CVD mortality varied by sex and by age. The other pair of compensating divergence and convergence components is on the regional axis itself. The cities of Moscow and Saint Petersburg are currently pioneering mortality improvements in Russia and are thus adding to the trend toward inter-regional divergence. This divergence effect is, however, compensated for by the trend toward convergence observed among the majority of the other Russian regions.

## Figures and Tables

**Table 2:**
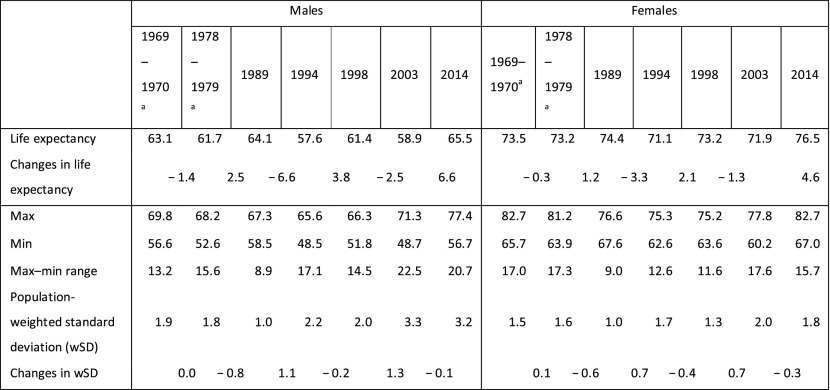
Population-weighted life expectancy at birth in Russia and some indicators of inter-regional disparities, selected years (in years). *Source*: Own calculation based on data provided by Rosstat

^a^The censuses of 1970 and 1979 were carried out in January. Therefore, population counts were produced not for the middle but for the beginning of the census year. To make death and population counts correspond to the same period, the sum of the regional death counts for 2 years (the year preceding the census and the census year) was divided by the doubled population estimated through the census. Thus, the mortality rates actually represent the mortality levels for a two-year period

## Appendix

**Table 1:** Cause-of-death groupings used in the analysis and their correspondence to cause-of-death classification systems

Cause of death	Russian Abridged Classification (1999) items	ICD-10 codes
Infectious diseases	1–55	A0-B99
Neoplasms	56–89	C00-D48
Cardiovascular diseases (CVD) + senility	115–147, 226	I00-I99, R54
Respiratory diseases	148–164	J00-J99
Digestive diseases	165–179	K00-K99
External causes	239–274	V01-Y98
All other causes	90–114, 180–225, 227	E00-H95, L00-Q99, R95

Ill-defined codes were redistributed as follows: (1) all deaths coded as “senility” (R54) were added to the “cardiovascular diseases” group because previous studies have shown that deaths from cardiovascular diseases after age 80 in Russia (and in some other post-Soviet countries) are often misdiagnosed as “senility” (Meslé et al. 1996; Shkolnikov et al. 1996; Grigoriev et al. 2012; Meslé and Vallin 2012). (2) All deaths coded as other ill-defined items (codes R00–R53, R55–R94, R96–R99 in ICD-10) were redistributed proportionally across all other groups of causes of death. There is a very strong negative correlation between the share of deaths coded as “senility” and the share of deaths coded as “circulatory diseases” (with the Pearson’s coefficient of − 0.95), whereas the correlation coefficients between the shares of “senility” deaths and of deaths from other groups of causes were much lower and were often insignificant (Danilova 2015; Vishnevsky et al. 2016).

**Table 3:** Year of the beginning of the recent life expectancy increase and its size, by sex and regions of Russia *Source*: Own calculation based on data provided by Rosstat

	Males					Females					Population size, mln people (2014)
	Year of the lowest life expectancy since 1998 (Y0)	Life expectancy		Life expectancy increase		Year of the lowest life expectancy since 1998 (Y0)	Life expectancy		Life expectancy increase		
		In year Y0 (years)	In 2014 (years)	Absolute (years)	Relative (%)		In year Y0 (years)	In 2014 (years)	Absolute (years)	Relative (%)	
Russia	2003	58.5	65.2	6.7	11.4	2004	71.8	76.4	4.6	6.4	143.8
Altai kray	2005	58.2	64.3	6.1	10.4	2006	71.6	75.7	4.0	5.6	2.4
Krasnodar kray	2002	61.0	67.1	6.1	9.9	2001	73.3	77.1	3.8	5.2	5.4
Krasnoyarsk kray	2000	56.0	63.5	7.5	13.4	2004	69.7	74.8	5.1	7.3	2.9
Primorsky kray	2005	56.8	63.3	6.5	11.5	2004	69.5	74.3	4.8	6.8	1.9
Stavropol kray	2003	61.0	67.8	6.8	11.1	2004	73.2	77.3	4.1	5.7	2.8
Khabarovsk kray	2005	55.3	62.2	6.9	12.5	2004	69.1	73.9	4.8	7.0	1.3
Amur oblast	2005	53.9	61.3	7.4	13.7	2005	67.5	73.0	5.5	8.1	0.8
Arkhangelsk oblast	2003	55.4	64.1	8.8	15.8	2004	69.9	76.3	6.4	9.2	1.2
Astrakhan oblast	2001	58.3	65.4	7.1	12.2	2003	72.1	75.9	3.8	5.3	1.0
Belgorod oblast	2001	61.2	66.8	5.6	9.1	2002	73.9	77.4	3.4	4.7	1.5
Bryansk oblast	2005	56.0	63.0	6.9	12.4	2003	71.7	75.9	4.2	5.9	1.2
Vladimir oblast	2002	55.1	62.9	7.7	14.0	2003	70.6	75.5	4.9	6.9	1.4
Volgograd oblast	2001	59.8	66.0	6.2	10.3	2000	72.8	77.0	4.1	5.7	2.6
Vologda oblast	2003	55.3	63.6	8.3	15.0	2004	70.7	75.8	5.1	7.2	1.2
Voronezh oblast	2002	59.1	64.6	5.5	9.3	2004	73.3	77.0	3.7	5.1	2.3
Nizhny Novgorod oblast	2005	56.4	63.2	6.8	12.1	2004	71.3	75.6	4.3	6.1	3.3
Ivanovo oblast	2002	55.0	63.7	8.7	15.8	2003	69.8	75.6	5.8	8.3	1.0
Irkutsk oblast	2005	53.3	60.5	7.2	13.6	2004	68.3	73.3	5.0	7.3	2.4
Republic of Ingushetia	1998	62.7	76.3	13.6	21.7	1999	73.2	82.0	8.7	11.9	0.5
Kaliningrad oblast	2005	54.9	64.7	9.8	17.9	2005	68.7	75.5	6.8	9.8	1.0
Tver oblast	2003	54.2	62.2	8.1	14.9	2003	69.2	74.6	5.4	7.8	1.3
Kaluga oblast	2005	57.5	63.3	5.8	10.2	2001	71.5	76.7	5.2	7.3	1.0
Kamchatka kray	2003	57.5	62.8	5.3	9.2	2002	68.6	73.8	5.1	7.5	0.3
Kemerovo oblast	2005	54.9	61.6	6.7	12.2	2006	68.8	74.0	5.2	7.5	2.7
Kirov oblast	2003	57.1	64.4	7.3	12.8	2004	71.0	76.8	5.8	8.2	1.3
Kostroma oblast	2003	55.3	64.0	8.7	15.8	2004	70.2	75.8	5.6	8.0	0.7
Samara oblast	2000	57.5	63.3	5.8	10.1	2001	72.2	75.8	3.6	5.0	3.2
Kurgan oblast	2003	56.9	62.4	5.5	9.7	2004	71.2	75.1	3.9	5.5	0.9
Kursk oblast	2005	58.0	63.7	5.7	9.9	2004	72.4	76.5	4.1	5.7	1.1
Saint Petersburg	2000	60.4	69.8	9.4	15.5	2001	73.0	78.6	5.6	7.7	5.2
Leningrad oblast	2003	54.9	64.7	9.8	17.8	2004	69.3	75.8	6.4	9.3	1.8
Lipetzk oblast	2003	58.9	64.4	5.5	9.4	2004	73.2	76.5	3.4	4.6	1.2
Magadan oblast	2000	55.7	61.6	5.9	10.5	2000	68.8	73.3	4.5	6.5	0.1
Moscow	2001	64.4	72.7	8.3	12.9	1999	74.5	80.3	5.8	7.8	12.2
Moscow oblast	2001	58.2	65.2	7.0	12.1	2003	72.1	76.3	4.2	5.9	7.2
Murmansk oblast	2003	56.8	64.0	7.1	12.6	2004	70.0	75.7	5.6	8.0	0.8
Novgorod oblast	2003	53.6	62.2	8.7	16.2	2004	69.1	74.5	5.3	7.7	0.6
Novosibirsk oblast	2005	58.5	64.4	5.9	10.0	2003	72.0	76.1	4.1	5.7	2.7
Omsk oblast	2005	58.6	64.0	5.4	9.3	2006	72.1	76.0	3.9	5.4	2.0
Orenburg oblast	2000	58.3	62.7	4.4	7.6	2006	71.9	74.8	2.9	4.1	2.0
Oryol oblast	2005	58.0	63.2	5.2	8.9	2003	72.4	76.6	4.1	5.7	0.8
Penza oblast	2005	58.7	65.6	6.9	11.8	2004	72.8	77.4	4.6	6.3	1.4
Perm kray	2003	55.6	63.1	7.5	13.5	2004	69.2	74.7	5.5	8.0	2.6
Pskov oblast	2003	53.7	62.1	8.4	15.6	2006	68.1	74.2	6.1	8.9	0.7
Rostov oblast	2003	60.4	66.0	5.6	9.3	2000	72.5	76.3	3.7	5.2	4.2
Ryazan oblast	2003	56.1	64.7	8.6	15.4	2004	72.1	76.7	4.7	6.5	1.1
Saratov oblast	2000	58.4	65.2	6.8	11.6	2004	72.1	76.4	4.3	5.9	2.5
Sakhalin oblast	2005	54.4	62.2	7.7	14.2	2006	68.0	74.0	6.0	8.8	0.5
Sverdlovsk oblast	2000	57.1	63.6	6.6	11.5	2004	70.8	75.6	4.8	6.8	4.3
Smolensk oblast	2002	54.7	63.3	8.6	15.8	2003	70.0	75.5	5.5	7.8	1.0
Tambov oblast	2000	58.0	65.2	7.2	12.4	2000	72.5	76.9	4.4	6.0	1.1
Tomsk oblast	2003	58.1	64.9	6.7	11.6	2004	71.1	76.4	5.2	7.4	1.1
Tula oblast	2003	55.9	63.5	7.6	13.6	2004	70.4	75.5	5.1	7.3	1.5
Tyumen oblast	2001	59.7	66.1	6.4	10.7	2003	72.5	76.7	4.2	5.8	3.6
Ulyanovsk oblast	2003	58.3	64.4	6.1	10.5	2003	72.2	76.1	4.0	5.5	1.3
Chelyabinsk oblast	2003	58.1	63.5	5.5	9.4	2004	71.4	75.7	4.3	6.0	3.5
Zabaikalsk kray	2005	52.8	61.6	8.8	16.7	2005	66.7	73.3	6.6	9.9	1.1
Chukchi autonomous area	2001	51.5	58.8	7.4	14.3	2002	61.2	67.4	6.2	10.1	0.1
Yaroslavl oblast	2003	55.8	64.1	8.4	15.0	2003	71.3	76.8	5.5	7.8	1.3
Republic of Adygeya	2003	61.4	66.8	5.4	8.7	2001	73.3	76.9	3.6	4.9	0.4
Republic of Bashkortostan	2003	59.8	63.7	3.9	6.5	2003	72.9	75.9	3.0	4.2	4.1
Republic of Buryatia	2003	54.4	62.7	8.3	15.2	2003	68.4	74.5	6.1	8.9	1.0
Republic of Dagestan	1999	66.1	72.5	6.5	9.8	1999	74.7	78.9	4.1	5.5	3.0
Kabardian-Balkar Republic	2005	62.8	69.3	6.5	10.4	2003	74.7	78.6	3.9	5.2	0.9
Republic of Altai	2003	54.0	61.7	7.7	14.3	2004	66.9	73.8	6.9	10.3	0.2
Republic of Kalmykia	2002	59.3	67.0	7.7	13.0	2003	71.6	77.0	5.4	7.5	0.3
Republic of Karelia	2003	53.6	63.0	9.4	17.5	2004	69.0	75.6	6.6	9.6	0.6
Republic of Komi	2003	55.4	63.1	7.7	13.9	2004	68.7	75.1	6.5	9.4	0.9
Republic of Mariy El	2004	56.7	62.8	6.1	10.8	2003	70.9	76.2	5.3	7.5	0.7
Republic of Mordovia	2003	59.7	65.2	5.5	9.2	2004	73.0	77.6	4.6	6.3	0.8
Republic of North Ossetia-Alania	1999	61.6	68.6	7.0	11.4	1999	74.4	78.4	4.0	5.3	0.7
Karachaev-Chercassian Republic	2003	62.2	69.0	6.8	10.9	2001	73.6	78.3	4.7	6.4	0.5
Republic of Tatarstan	2004	60.8	66.3	5.5	9.1	2001	74.4	77.7	3.3	4.4	3.8
Republic of Tuva	2002	48.2	56.7	8.5	17.6	2003	60.2	67.2	7.1	11.7	0.3
Udmurt Republic	2003	57.4	63.5	6.1	10.6	2003	71.1	76.4	5.2	7.4	1.5
Republic of Khakasia	2003	54.3	63.0	8.7	16.1	2004	67.6	74.6	7.0	10.4	0.5
Chechen Republic	2003	63.8	70.1	6.3	9.9	2011	74.6	76.0	1.3	1.8	1.4
Chuvash Republic	2002	59.5	64.4	4.9	8.3	2003	72.6	76.9	4.3	5.9	1.2
Republic of Sakha (Yakutia)	2001	57.3	64.3	7.0	12.2	2000	69.9	75.5	5.6	8.0	1.0
Jewish autonomous oblast	2005	53.7	59.5	5.8	10.8	2006	65.7	71.4	5.7	8.6	0.2

**Table 4:** Decomposition of the mortality-related part of the life expectancy increase by the regions of Russia, age groups, and sex, 2003–2014. *Source*: Own calculation based on data provided by Rosstat

	Males				Females			
	Total (years)	Including by age groups (%)			Total (years)	Including by age groups (%)		
		0–14	15–59	60+		0–14	15–59	60+
Total	6.46	9.3	63.3	27.5	4.44	11.2	36.1	52.8
Moscow^a^	0.61	8.5	42.7	48.8	0.41	8.7	27.5	63.8
Moscow oblast	0.31	8.2	66.7	25.1	0.20	11.9	44.5	43.6
Saint Petersburg	0.29	4.9	66.4	28.7	0.19	6.5	42.1	51.3
Krasnodar kray	0.21	7.7	63.0	29.3	0.13	10.4	31.7	57.8
Sverdlovsk oblast	0.19	10.4	65.9	23.7	0.15	13.6	34.8	51.6
Rostov oblast	0.17	9.5	62.2	28.3	0.11	16.6	33.1	50.3
Nizhny Novgorod oblast	0.15	9.5	67.3	23.2	0.10	14.4	34.7	50.9
Krasnoyarsk kray	0.15	9.2	71.8	19.0	0.10	9.9	43.0	47.1
Tyumen oblast	0.14	10.1	57.7	32.2	0.10	12.0	29.0	59.0
Perm kray	0.14	8.6	70.2	21.2	0.10	11.3	40.2	48.6
Republic of Tatarstan	0.14	11.4	62.4	26.2	0.08	10.5	26.6	62.9
Chelyabinsk oblast	0.13	10.0	63.1	26.9	0.11	12.7	29.5	57.8
Stavropol kray	0.13	3.4	66.7	29.9	0.08	4.0	39.8	56.3
Kemerovo oblast	0.13	10.4	68.2	21.4	0.10	8.9	40.9	50.2
Irkutsk oblast	0.12	10.3	71.9	17.8	0.09	11.8	40.4	47.8
Leningrad oblast	0.12	3.9	74.1	22.0	0.08	5.2	51.1	43.7
Republic of Bashkortostan	0.11	13.8	56.7	29.5	0.08	16.4	18.2	65.3
Saratov oblast	0.11	11.9	61.8	26.2	0.08	14.6	29.0	56.4
Volgograd oblast	0.11	11.6	62.5	25.8	0.07	12.2	34.3	53.5
Novosibirsk oblast	0.10	12.3	63.2	24.5	0.07	12.2	37.0	50.7
Samara oblast	0.10	9.7	52.4	37.9	0.07	11.8	19.0	69.3
Primorsky kray	0.09	13.6	62.5	23.8	0.06	21.3	34.9	43.8
Republic of Dagestan	0.08	13.5	43.0	43.5	0.05	11.4	33.6	55.1
Altai kray	0.08	10.6	60.9	28.5	0.06	6.7	30.7	62.6
Voronezh oblast	0.08	9.2	60.4	30.5	0.06	15.9	30.8	53.2
Tula oblast	0.08	6.4	71.3	22.2	0.06	8.9	43.8	47.3
Arkhangelsk oblast	0.08	6.5	73.2	20.3	0.06	9.1	42.6	48.3
Tver oblast	0.08	9.2	70.7	20.1	0.05	7.4	48.1	44.5
Yaroslavl oblast	0.07	7.2	71.3	21.4	0.05	9.2	46.3	44.5
Vladimir oblast	0.07	6.3	73.4	20.3	0.05	5.5	46.1	48.4
Kirov oblast	0.07	9.3	66.0	24.6	0.06	13.7	35.8	50.5
Vologda oblast	0.07	7.5	74.5	18.0	0.04	12.5	39.6	47.9
Ryazan oblast	0.07	11.3	69.6	19.1	0.04	12.2	37.7	50.2
Khabarovsk kray	0.07	10.4	67.9	21.7	0.04	11.1	41.1	47.8
Zabaikalsk kray	0.07	11.5	68.7	19.8	0.04	12.8	50.8	36.4
Penza oblast	0.07	13.4	65.4	21.2	0.05	17.9	33.4	48.7
Kaliningrad oblast	0.07	8.9	72.6	18.5	0.04	9.9	51.8	38.3
Udmurt Republic	0.06	7.5	67.6	25.0	0.05	10.1	31.5	58.3
Omsk oblast	0.06	12.6	55.7	31.6	0.05	16.8	27.2	56.0
Ivanovo oblast	0.06	8.1	73.5	18.4	0.04	12.5	42.1	45.5
Republic of Buryatia	0.06	10.5	71.8	17.7	0.04	12.9	42.5	44.6
Orenburg oblast	0.06	15.2	54.2	30.7	0.04	13.2	23.5	63.3
Chechen Republic	0.06	1.1	80.9	18.1	0.01	− 7.4	111.4	− 4.0
Smolensk oblast	0.06	7.1	70.3	22.6	0.04	15.3	37.8	47.0
Ulyanovsk oblast	0.06	8.2	65.5	26.3	0.04	11.6	25.3	63.1
Tambov oblast	0.05	9.2	67.7	23.1	0.03	18.3	26.7	55.0
Republic of Komi	0.05	6.3	70.5	23.2	0.04	6.6	46.0	47.4
Belgorod oblast	0.05	9.5	60.6	29.9	0.03	10.8	36.9	52.3
Bryansk oblast	0.05	10.2	65.4	24.4	0.03	8.6	36.5	54.9
Tomsk oblast	0.05	18.8	53.8	27.4	0.04	18.1	35.7	46.2
Astrakhan oblast	0.05	8.0	62.3	29.7	0.03	8.3	32.7	59.0
Lipetzk oblast	0.04	4.7	67.0	28.3	0.03	10.7	29.5	59.8
Chuvash Republic	0.04	12.8	57.7	29.5	0.04	9.7	27.9	62.5
Republic of Karelia	0.04	4.8	75.2	20.1	0.03	3.3	48.9	47.9
Republic of Sakha (Yakutia)	0.04	13.7	61.6	24.7	0.03	9.9	32.9	57.2
Murmansk oblast	0.04	3.9	69.1	27.0	0.03	5.0	42.3	52.7
Kostroma oblast	0.04	7.4	74.5	18.1	0.03	13.0	42.5	44.5
Kursk oblast	0.04	10.2	64.4	25.4	0.03	15.2	37.9	46.9
Pskov oblast	0.04	15.2	65.5	19.3	0.03	13.1	47.3	39.6
Kabardian-Balkar Republic	0.04	12.1	50.1	37.8	0.02	17.0	30.8	52.2
Kaluga oblast	0.04	7.8	65.2	27.0	0.04	8.6	41.0	50.4
Amur oblast	0.04	13.7	65.9	20.5	0.03	19.9	38.2	41.8
Novgorod oblast	0.04	9.8	71.6	18.5	0.02	7.5	42.6	49.9
Kurgan oblast	0.04	15.5	57.6	26.9	0.03	15.4	27.4	57.2
Republic of Khakasia	0.03	13.2	69.1	17.7	0.03	9.6	50.4	40.0
Republic of Mordovia	0.03	4.1	69.8	26.1	0.03	16.9	30.8	52.3
Republic of North Ossetia-Alania	0.03	6.3	62.0	31.7	0.02	− 2.4	36.4	66.0
Oryol oblast	0.03	8.7	68.3	23.0	0.02	11.3	37.0	51.7
Republic of Mariy El	0.03	10.2	65.7	24.1	0.02	10.2	29.4	60.4
Sakhalin oblast	0.03	11.6	60.7	27.7	0.02	7.3	44.2	48.5
Karachaev-Chercassian Republic	0.02	9.5	55.3	35.2	0.01	4.3	36.7	58.9
Republic of Tuva	0.02	15.4	72.2	12.4	0.01	11.5	56.8	31.7
Republic of Adygeya	0.02	8.5	66.7	24.8	0.01	4.4	37.3	58.3
Republic of Ingushetia	0.02	15.4	41.5	43.1	0.01	22.6	13.7	63.6
Kamchatka kray	0.01	11.4	60.3	28.3	0.01	7.9	36.3	55.7
Republic of Kalmykia	0.01	8.0	66.5	25.5	0.01	13.0	42.8	44.2
Republic of Altai	0.01	18.7	66.2	15.1	0.01	11.6	43.4	45.0
Jewish autonomous oblast	0.01	20.6	68.0	11.5	0.00	− 0.9	50.7	50.3
Magadan oblast	0.01	9.7	31.5	58.8	0.00	16.8	7.7	75.5
Chukchi autonomous area	0.00	41.0	41.1	17.8	0.00	19.2	12.1	68.7

^a^Regions are listed in the descending order by their contribution to the total change in life expectancy

## Footnotes

1 Before 1990, there were 73 regions at the highest level of Russia’s administrative division. After the dissolution of the Soviet Union, a few of these regions were split into smaller units, which led to an increase in the total number of regions from 73 to 79 in 1990, and from 79 to 83 in 1993. Three of these units remained under the jurisdiction of other regions while still being classified as separate regions; thus, Rosstat publishes statistics for the Tyumen region that includes the Khanty-Mansy and the Yamalo-Nenets autonomous districts, and for the Arkhangelsk oblast that includes the Nenets autonomous district. Following Rosstat, we distinguish between 80 regions in our study. Since 2014, Rosstat has also been collecting and publishing data for the Republic of Crimea and the city of Sevastopol, which formed two new entities of the official administrative division of the country. To ensure consistency with the earlier series, we do not include them in our present analysis. Along with the discrepancies caused by changes in administrative divisions, there are also a few gaps in the territorial coverage of the vital statistics. Death counts are not available for the Ingush Republic in 1993–1994 and for the Chechen Republic in 1993–2002.

2 Sweden is the country with the longest series of reliable mortality data. Swedish mortality series (at older ages in particular) often serve as the gold standard for assessing the quality of mortality statistics in other populations (Jdanov et al. 2005).

3 The death rates for the age group 85 and older (*m*_85_) were calculated as 1*/e*_85_, where *e*_85_ is the life expectancy at age 85 derived from complete life tables, with ages 100 + as the last open-ended interval.

4 The value of population-weighted average life expectancy may differ slightly from the value calculated for the population of Russia as a whole. For instance, in 2014 life expectancy at birth calculated for the whole population was 65.3 years for males and 76.5 years for females, while the population-weighted average life expectancy was 65.5 and 76.5 years, respectively. But as the two indicators are almost equal, we will continue to use the term “life expectancy” for the sake of simplicity.

5 While decomposing the changes in the inter-regional disparities, we assessed mortality and population composition parts (M- and P-effects). As soon as the age-specific M-effects are much greater than the P-effects, we consider the M-effects only.

6 In 2012, the city of Moscow was enlarged by a territorial expansion into the Moscow oblast. Due to the inclusion of some rural areas in the city’s borders, 1.5% of the population of the city of Moscow is regarded as rural in the statistics. In the city of Saint Petersburg, 100% of the population is regarded as urban in the statistics.
